# A Criticism of the “Germ Theory of Disease,” Based on the Baconian Method1Read before the Birmingham and Midland Branch of the British Medical Association, October 11, 1894.

**Published:** 1894-12

**Authors:** Lawson Tait

**Affiliations:** Consulting Surgeon to the Hospitals for Women at Birmingham, Nottingham and Southampton, and to the West Bromwich Hospital, etc.


Buffalo Medical and Surgical Journal

VOL. XXXIV. DECEMBER, 1894. No. 5.

### Original Communications.

### A CRITICISM OF THE "GERM THEORY OF DISEASE," BASED ON THE BACONIAN METHOD.[1]

BY LAWSON TAIT, F. R. C. S., etc., etc.,

Consulting Surgeon to the Hospitals for Women at Birmingham, Nottingham and Southampton, and to the West Bromwich Hospital, etc.

SOME ten years ago, and lasting for about six years, a revival occurred of what has unfortunately appeared in various forms too often, and from which we cannot expect to escape altogether in the future, the employment of electricity as a remedy for disease. In this instance it was directed to the treatment of affections in the female pelvis, and we were told that inflammatory effusions, purulent inclusions in the tubes and solid tumors in the uterus had melted away under the influences of electrolytic currents. My own criticism of the proposals were mainly confined to the suggestion that as inflammatory processes were pretty much the same in all particulars wherever met with, I should be content to believe the statements and follow out the treatment if I saw it applied successfully to the products and results of a whitlow or a ganglion of the wrist.

My suggestions were not listened to; at least I have never heard that they were carried out successfully, and the electrolytic treatment in gynecology has already gone the road of all such things, after doing infinite harm in all directions save one.

In this room some two months ago two papers were read, to one of which I listened with the greatest care, for it was on a subject concerning which I had no practical knowledge, though I had read much and with great interest on the results of researches in bacteriology—the subject of Dr. Armand Ruffer's paper. Only

1. Read before the Birmingham and Midland Branch of the British Medical Association, October 11, 1894.

one thing in the paper struck me with dissatisfaction, and that was the constant reiteration of expressions by the author of the perfect certainty of the conclusions he asserted. I took part in the debate, and mildly suggested that its cock-suredness was possibly a little premature. Now I am certain of it, for I have followed in my reading with a growing conviction that the same offer of criticism may be made to it as I made in the case of pelvic electrolysis. Let us see how its assertions bear the test of examination by the great broad lines of disease, and by the phenomena of such diseases as place their leading features under our eyes for inspection, diseases upon the course and distribution of which we can gather exact and trustworthy information, diseases in the course of which we can perform experiments entirely legitimate and under logical conditions—in fact, diseases where we can argue without the probability of leaving out altogether the major propositions of our syllogisms, or of entirely neglecting what may be really the *causa causans*.

I entered my college career just as the grand sweep of the cellular pathology was careering over the medical world. Everybody was mad after a new cell. Special courses and special teachers were told off for cellular instruction. Our microscopes had got so far as to unriddle the facts of cellular construction, and therefore there must be a cellular pathology which was to explain everything. Two parties existed and of course they fought bitterly, the only real and permanent effect being that the unhappy student had to meet two sets of examiners and to know two sets of answers to the same questions, the one to be given as the wind blew from the east, the other as the turn of the west wind came. The cellular party apparently triumphed, but it has silently and mysteriously disappeared, so that we may ask with Hans Breitman, "where is dad barty now?"

When we looked down our brass tubes at pathological products we saw, so clumsy were the sections, masses of cells so indefinite that they might have been slices of cheese, but we believed and we wondered accordingly. Here and there we saw curious little bright points in quivering movement, and when asked about them we answered that they were "vibrating molecules." A molecular pathology grew up, but died with its one exponent.

During the twelve years in which I was a hardworking and enthusiastic microscopist the process of staining was developed with great advantage. And here I want to indulge in a little bit of egotism, and remind those persons who now sneer at the views of a mere practitioner like myself as unscientific, and the person himself as one of no scientific attainments, that one of the most selective of all methods of research by staining was my own invention ; that I was the first to show that fresh tissues of the greatest delicacy, such as the umbilical cord, could be cut of less than a thousandth of an inch in thickness so perfectly that the whole diameter of the cord could be cut and mounted fresh without a single air bubble in it. In this way I unraveled the anatomy of the cord so completely that not a single fact has been added to or taken from my description during the eighteen years which have elapsed. But with the introduction of immersion lenses my work at the microscope ceased, for I found something better to engage me.

Since then the extension of our powers of sight and our methods of staining have resolved our "vibrating molecules" into a vast array of little beasts, about which thousands of facts of the greatest interests have been discovered—but about which the facts have been misconstrued, alike in importance and in direction. From some of these facts another great theory by which everything is to be explained, and under which everything is to be squeezed, has been promulgated : it is called the "Germ Theory" and its modern, or rather its most recent developments, I propose to take as my subject of this present paper. I am the more induced to do so, in that my old friend and former pupil, whom I honor in his place today as our President, and congratulate on that well-merited distinction, took the same question, and the same title as the subject of his Presidential address. I liked the address very much, and I fully appreciated the gracefully concealed, but quite as graciously apparent things he said about myself ; not only so much for the things he said, as the kindly way he said them, I thank him heartily.

I therefore sincerely hope he will forgive me, when at the very beginning of what I have to say, I declare his title to be entirely mistaken.

Throughout the range of all branches of medicine and surgery one of the greatest of its defects is its nomenclature, a statement which is proved by the constant, meaningless and purposeless changes we make in it. If there is any defect greater than this in medical literature, it is its defective logic—its logical processes are generally so bad that its writers might never have had any experience whatever in the simple equation of algebra or the first book of Euclid, though these are the very best trainings in logic which the world is possessed of.

Let me remind you, that not only all the canons of logic, but all the facts of discovery recognize three stages of generalization, the hypothesis, the theory, and the law.

The hypothesis is represented by the first statement of an algebraic equation—let $x$ equal the unknown quantity; and then all the facts concerning $x$ are twisted and turned about on both sides of the sheet till it is shown either that $x$ is an ascertainable quantity, or that the equation problem is faulty by reason of some inaccuracy or some deficiency in the statement of the case.

The theory, as a development of the hypothesis, is well seen in most of the problems of the first book of Euclid, as may be stated in that where it is offered for our belief, that if two triangles are equal in one side and in one angle they must be equal in all other respects. That being established by the *reductio ad impossibile* the conclusion becomes a law. If it could be disputed by a single example, it would no longer be a law, as we can see that it would be by countless problems suggested by the possibility of their being a fourth dimension in space—as for example, that the nearest route between two points need not necessarily be a straight line. Indeed, the establishment of a fourth dimension in space would make Euclid's problems a series of amusing hypotheses, pretty much what the so-called germ theory of disease is now, with a series of interesting facts quite incapable of coördination, or of practical application. And I must ask you to bear in mind that there are a great number of very competent people who believe that this supposition has been proved. Ledocowski's suggestion that parallel lines may really approach one another, yet never meet, is one of the many points by which eminent mathematicians regard it even now as a workable theory.

The use of the word theory is so much a favorite pastime in medical literature, that to say that it occurs generally abused as an undistributed middle term, is a mere flippancy.

The abuse of the word is a notable example of the utter absence from our common professional language of any sustained effort to respect the first essential of logic, constant and respectful definition. We speak with equal complacency of the theory of some lunatic patient that the moon is made of green cheese, and of the kinetic theory of gases. The first has not a single fact on which it is based, while the second has explained the solar atmosphere, the total absence of hydrogen and oxygen from ours, and the fact that on the invincible union of hydrogen and oxygen the whole economy of our universe depends.

The kinetic theory has almost become the law of gaseous motion. The germ theory of disease is not a theory at all. It is not even a hypothesis. It is a mere congeries of facts some truly stated, but mixed up with a far greater bulk inaccurately (indeed, untruly stated), upon which not even a working hypotheses has yet been suggested, far less a theory built.

From the earlier doings of humanity the farmyard science and the roughest domestic medicine recognized the fact that diseases of certain and familiar kinds were conveyed about by poison.

The belief concerning an exanthematic disease or plague, which commended itself most to the ancient Hebrews, and to the Greeks, was that it was a blow from an offended or malevolent deity.

Under the new dispensation the favorite Christian doctrine has been that it was a visitation from the devil. For both the modern scientist has substituted a microbe. But this is no theory, it is not even an advance in logical conclusion or construction over the Jew of Mosaic times.

Any one who will read attentively (as I fear very few do now-a-days) the Book of Leviticus, especially the latter half of the 14th chapter, will see that the fact of the conveyance of disease by a poison was completely recognized by its author. The ancient Jews were a very practical, business-like, and honest people, and could have written sanitary articles in medical journals much better than some of their descendants of the present day do now.

They knew the facts of sepsis, and had most elaborate schemes of antisepsis, particularly for the gonococcus; and their stringent antiseptic details were not much more ridiculous, or probably much less satisfactory than many of the myriads that have emanated from Lister himself.

The facts of the poison origin of certain diseases has *always* been accepted, but the suggestion of the immediate machinery has, as I have shown, curiously varied. The last suggestion is that of the microbe—he may be displaced by a "smaller flea"; but even if he proves to be the ultimate fact, he is only a fact, nothing more than a fact, certainly not a theory.

The road by which we went astray in this matter was one that might have been set as a trap. It began in the famous discussion between Pouchet and Pasteur on biogenesis. Pouchet believed, erroneously, that he had discovered life in its cradle, whilst Pasteur made the far more practical discovery of the ultimate (or probably ultimate) facts of decomposition. He did not invent a hypothesis, he did not formulate a theory, and he did not establish a law—all that had been done before—he simply added facts.

The law of decomposition had long been known; given a solution of organic compounds, maintained at a certain temperature to which the air has access, and they will decompose. Pasteur (and by that name in this research I indicate a host of other names of whom many are perhaps equally important) added the fact that the process was always in the presence of, and on account of the presence of numerous minute organisms having sexual arrangements (unless these had parasitically suppressed) and producing spores. But how absurd it would be to call this contribution of facts, by Pasteur (and others) the formulation of a theory, or the establishment of a law.

Let me here remind you of the essence of inductive reasoning in Bacon's own words—a rule which has never been successfully evaded:

"The form which is sought can be detected only by a process of exclusion, by which we find a phenomenon constantly present when the effect is present, absent whenever the effect is absent, and varying in degree with the effect. Such a phenomenon would be the form in question, the cause of the given effect or attribute."

According to this salient definition is the *causa causans* of decomposition the microbe? Most certainly not. Therefore, we can have no microbe theory of decomposition. The reason is that in the application of the Baconian method we find there are two conditions under which the microbic influence is futile. The first, such a state of matters as may be fully illustrated by the homely instance of well-made jam—an illustration full of the most intense interest, when studied in the light of recent facts of bacteriological research.

The second and constant exception is when the solution of organic compounds maintained at a certain temperature with free access of air is at the same time endowed with *life*. The most delicate organic compound thus endowed, known to me, is the medusa. So long as it can maintain its rhythmic movements it can defy the germ spores and the fully developed animals whose work is decomposition. Let these rhythmic movements cease and they remove all trace of it in a time which may almost be stated in minutes. The processes of decomposition and disease are, therefore, not the same, as some of the earliest and wildest of the germ theorists boldly asserted. These were chiefly the surgical antisepticists who attributed to *all* germs *everywhere* a powerful pathogenic influence; and who, therefore, devoted much ingenuity, and all their energies, to make their conduct harmonize with this belief, by sprays, veils, douches, etc., etc. A few of them still remain.

But if we proceed on true Baconian lines we find that not only are the phenomenon of decomposition not those of disease, but that there is absolutely no analogy between them. Some appearances of analogy there are, but they are easily destroyed by careful examination. Thus, take the huge carcass of an ox, let it lie in the field till the phenomena of decomposition are observed beneath the eyelids. At the same time they will be found active in the pericardium, though all possible communication by blood current between these two such remote spots has been destroyed.

The conclusion from the facts above is inevitable—that the spores of the animalcules of decomposition are always present in our bodies, and in the bodies of animals, and that they commence their victory at once after death (and probably not unfrequently before it): How else can we explain the familiar phenomenon of a piece of meat half cooked to make it keep going putrid in the uncooked middle and keeping sweet in the cooked outside? This is a fact known in domestic science for all time, and did not need the elaborate researchers of the bacteriological school for its re-discovery. Many of these researchers would have been saved much trouble if they had studied for six months under a housewife before they went to the laboratory.

Throughout the whole nomenclature of disease I know of none which affects the whole mass of the living body at once and at a blow, as does decomposition.

Of course the sciolists will at once shout out "the exanthematic fevers," but for them the circulation of the blood makes the difference.

They are probably diseases of the blood as is shown by the modification of the functions, or even the entire suspension of the functions of the great viscera which is so preëminently characteristic of all of them. If it is the blood which is their nest the whole body is of course only *apparently* affected by the diffusion of that part of it *really* affected throughout its whole, and, therefore, the supposed analogy fails.

Again and again the analogy fails in directions which I cannot follow, partly for want of time, partly for want of knowledge of detail, so I content myself with such illustrations of the defect as variations of degree; selective attacks based apparently upon the most eccentric considerations or, rather want of them; the occurrence of epidemics, and countless other points which are wholly irreconcilable with there being any analogy with which the vagaries of exanthematic diseases and the hum-drum uniformity of the phenomena of decomposition in dead tissue.

There is another fact, or rather two, on this the negative side of the argument, not unworthy of the enquirer who has an open mind on this subject—and that person belongs to the small minority of the profession at the present moment. The first is the really small value to be attached to the great bulk of what the bacteriologists are pleased to call original research, owing to the flat contradictions they give to one another, a trenchant illustration of which I shall shortly give you.

The second is that it is quite possible that they are all putting the cart before the horse. It has been my business to follow the researchers on the bacillus of tubercle with great care, because I have objected to a group of cases of abdominal disease being designated "tuberculous peritonitis."

Opening and draining these cases cures the majority of them; and in the characteristic "tubercles" of a great many of my cases the "bacillus tuberculosis" has been found, whilst in others it has not. Many of the former have been amongst the recoveries, and some of the latter have been amongst the cases which ultimately died of the advance of the disease. Hence, I conclude that the characteristic bacillus of tubercle (about which I am sure there can be no doubt whatever that it exists) is a product of the disease and not its cause. The experience of abdominal surgery seems to me to admit of no doubt whatever on this point, and it is certainly supported by the facts of the symmetrical and selective location of the disease, of the enormous proportion of cases of it really and permanently cured, of its age periods, its symmetrical distribution, and many other points on which I should like to talk for hours. Not one single fact or observation in the vast array of literature on this subject goes even to support a hypothesis that the bacillus is a *causa causans* of the disease. If it were so how could any of us escape?

The whole of the phenomena of the tubercle bacillus, as they are known to me, are capable of a very simple explanation, and I advise some of the workers to start a research on this hypothesis: that the tubercle exudation is an unorganized material of feeble vitality (or perhaps like pus, of no vitality at all), and therefore capable of resisting the attacks of the ordinary bacilli of decomposition but feebly, and that the bacillus of tubercle is but a sport of some more ordinary form produced by a fresh soil. This sport may, by reason of constant feeding on appropriate soil, have become possessed, not only of specific form but of specific powers and finally a secondary habitat. All these suggestions are actual and well-known facts in the life history of many parasites—such as the fluke. Like the case of the fluke such a supposition would bring into order the climatic and localized increments of tubercle, as at Salisbury; its apparent contagiousness, and most of all its hereditary tendency, which is certainly not a fact that can be brought within the present microbic speculations.

I propose now to leave the side of negative argument—always a process full of difficulties and possibly never complete—and deal with a more positive battery of attack. I mentioned at the beginning of my paper the use of electrolysis in gynecology, and the suggestion I used that its powers should first be proved on diseases accessible to easy examination with indisputable results—such as a whitlow. I propose for the examination of this "germ theory" to look closely into an analogous position, and I have followed therefore with the most intense interest its development in the field of dermatology.

The skin doctors have speedily arranged themselves into two camps, germists and anti-germists. They have quarreled and slanged each other like gynecologists, and have settled nothing.

When it is seen that the great bulk of the phenomena of diseases of the skin are under their naked eyes, that these may be supplemented by simple, harmless and perfectly justifiable experiments and perfect optical appliances, it is most disappointing to find that the whole and sole results are numerous changes of nomenclature and the blatant advertisement of nostrums—some of them approaching quackery so closely that no other term is applicable to them.

Let me take one of the most common and, from the side of the germists the most simple of all skin diseases—eczema, a very common ailment about which there ought to be as little mystery and as little difference of opinion as about any possible disease. At the very outset the difference of opinion amongst dermatologists show upon what slender grounds the germists stand. They are not agreed as to whether it is of local or constitutional origin. At Bristol they had a field-day on this question ; the President of the section boldly declared for its origin in parasitic affection, all constitutional relations being entirely secondary. Dr. Unna of course declares in favor of microbes or the *causa causans*, the way for there being paved by a seborrhea. But the Dutch Professor forgot that the seborrhea itself is not without a *causa*, and must be explained as a preliminary.

Another school, led by Dr. Myrtle, is in favor of its neurotic origin, and declares that microbes have nothing to do with it. It is clear that if we cannot settle this question in the case of eczema, there are few cases where it is capable of settlement.

But there are many facts from which we may draw side lights, and by the application of the Baconian system of reasoning show that it is impossible to accept the microbic origin of the disease.

First of all it is clearly selective in the great bulk of cases, that is, it takes up its abode in certain parts of the body, its extent is limited, often very strangely, as at the back of one finger, and it is very often absolutely symmetrical.

It must be quite apparent that every disease which is selective must have its *causa*—that is, its governing *causa*, either in the nature of the tissue affected, or in the innervation of the tissues. As the skin is uniform, or practically so, in its construction, it is only in the innervation of the skin that the peculiar selective characters of eczema can be explained ; and the strongly marked tendency toward symmetrical arrangements of disease are capable of an explanation through the nervous system, and that only. Mere blood channels cannot explain it.

Eczema is not capable of being conveyed from a diseased person to a healthy one indefinitely. Even when it does appear, as it seems now to be appearing in London, in epidemic form, its appearance is strictly limited to those in whom defective nerve nutrition is most apparent.

Even, therefore, if all the authorities admitted the universal presence of the culpable microbe—and the authorities do nothing of the sort—according to Bacon's law the *causa causans* of eczema is not the germ, nor the microbe, but the soil in which it is permitted to grow, by the conditions into which that soil is permitted to pass by some real cause over which it is probable the nerve centers have control. The germ is a mere agent, at the best one of many factors and by no means the chief.

That I have pressed, and press to the uttermost, the initial difficulty of the application of the phenomena of germ decomposition in substance already dead, might begin to weary you, were it not that fresh light can be brought to bear upon the subject from tissues that have passed from life to death, but still remain attached to the body—cases of epithelial strictures like the hair. The hair possesses no phenomena of life, apart from its point of growth, yet it has a pathology of the deepest interest; and I read Dr. Leslie Roberts' paper in a recent number of the *British Medical Journal* on the pathology of vegetable hair parasites with the extremest interest, because it was a phase of the dermatological investigations getting as near the laboratory flasks as seems possible. His first sentence, however, was so completely in harmony with my own previous rendering, that I feared I should get no fresh light. He says: "Considering the amount of research which has been given to the vegetable hair parasites since the days of Gruby, it is surprising how little progress has been made in the direction of real knowledge." And he goes on to show that, as far as results and methods of enquiry, they are no further advanced than botanists were in the times of Gussieu and Linnæus, and that the philosophical revolution affected by Darwin has not yet reached them. They have as yet got no further than species-mongering, and in this respect he speaks, and speaks most properly, of the scientific abuse of the method of culture as a method of research. I wish amongst the culture researchers of general pathology such another Solon would arise, and put an end to the multitude of drivel that is made to center round the culture tube. He says: "The scientific absurdity of this method becomes apparent if we suppose an analogous method adopted towards the higher plants. What should we say if an investigator provided to classify the trees in an orchard by the shape of their branches or the color displayed by their fruit? This logic, as De Bary says, does not change with the size of the objects or with the apparatus and manipulation." Further, Dr. Leslie Roberts says: "Even the successive memoirs are self-contradictory to some extent, and no wonder, for they appear with a rapidity which is unusual in scientific research, and suggest to the reader the necessity of reserving his judgment." "Many of the opinions maintained so strongly have passed into medical currency with very little enquiry on the part of those who have received them." All this is pretty strong language, but not half so strong as, in my opinion, the occasion justifies. I have not followed in practical criticism, as Dr. Roberts has done, the work of those to whose conclusions he raises his possible objections. But I have risen over and over again from the perusal of such papers as he names, with a feeling of the saddest kind, that this ephemeral nonsense is what passes for science with the bulk of the present members of my profession. It is carried on without regard to the first principles of Bacon, and with an utter disregard to the teachings and examples of Darwin. The very fundamental teachings of the evolutionary school of biology are brushed aside, and the great text-book of Sach's might as well never have been written.

By one sentence Dr. Leslie Roberts puts aside all the conclusions of the researches on hair parasites. "It has not hitherto been pointed out, so far as I know, that these fungi attack only domesticated animals; but this, I believe, is the truth." Here is one great fact which knocks the germ theory on the head with an insistance that the ground should be gone over again by someone who has a logical mind. Certainly without that we cannot accept a single conclusion of the germ hunters concerning skin parasites and that on a ground where the research would seem to be reduced to its most simple and least complicated form.

The fact is that we are clearly neglecting the main statement of our enquiries, and if we are ever to find the true answer we must make the first line, as in algebraic equation: Let $x =$ the condition of the soil in which it is possible for a spore or microbe to grow—even if we grant, for the sake of argument, that they can grow at all.

Let me make a little more clear what I mean by an example taken from natural history. It is perfectly well known to all observers of nature that within a very few seasons of the openings of subsoil by such a process as the cutting of a railway embankment, plants not previously known in the particular locality will spring up in abundance. Those of us who have ever made new gardens out of old turf turned up will remember the amazement at the kinds of new weeds which the digging and trenching brought up, to the very margin of the last spade hole, and the extraordinary vigor of growth of the first of the new comers.

In some cases, where careful research has been made in the case of railway cuttings, the new plants have not been found as old inhabitants within a hundred miles of the new surface. Would a germ theory suit this case? Certainly not! Did they wash out the new soil and carefully hunt for the germs and indefinitely breed them by culture experiments? Certainly not! They never troubled themselves. For a time there seems to have been a suggestion that the seeds were blown from a distance, or carried by birds, but this was speedily settled by the enormous number of plants which grew at once—far more than could be explained by seed blowing; and the phenomena were accepted to be due to the saturation of the soil and subsoil with seeds of plants of species which had long died out in the neighborhood, such seeds when properly protected having a vitality indefinitely prolonged. It would be difficult to understand a vast host of the phenomena of vegetation save for this hypothesis.

The *causa causans*, then, in our phenomena of the fresh clothing of the railway embankment is the turning up of the soil. It is the one cause without which all others are of no avail; without this new condition of the soil those seeds would have remained dormant, as have done the seeds of the reeds and palms of the coal measures, till they died, to be seen again only as geological specimens in the cabinets of future paleontologists.

The favorite hunting ground for the modern microbe catcher is a disease which presents the phenomena which have been very properly and, for my purpose, very aptly termed zymotic. There is a tendency to drop this most useful word, and I have no desire to argue for its retention save my dislike to those constant, unnecessary and most misleading changes. "Zymosis" is quite as false as any of the old or as the new attempts at definition. It arose from the rough analogy which was drawn between the rhythmical phenomena of the brewer's vat, with its exaltation of temperature, and the conditions of certain diseases. Surely amongst these diseases the germ theory ought to be established? My answer is, most certainly not; among these least of all, and for many reasons, though mainly for one. Take a hundred vats, with their solutions of sucrose, glucose, diastase or starch, and drop in your germ and you will get results absolutely answering to Bacon's law, "the phenomena constantly present when the effect is present, absent when the effect is absent, and varying in degree with the effect." Pasteur's wonderful elucidation of this law in the process of brewing and wine fermentation, and the enormous financial benefits which he conferred on those trades were the cause of his leading so wildly astray the young doctors who had never studied Bacon and were incapable of grasping the fact that whilst Pasteur might be wholly right in the brewing vat, it required a re-arrangement of the syllogism to carry the argument to the human body. Take 10,000 human bodies all perfectly alike, as far as we can see, and drop your germs of typhus about and see what will happen. The first remarkable fact to be found is that if the bodies are in Birmingham there will probably be no effect at all. If they are in Edinburgh no effect, or very little will be seen in the new town; whereas in the old town some commotion will result and there will be about a hundred cases of typhus. These will be distributed in various centers with mathematical proportion, and probably no two cases will be exactly alike. Clearly, then, according to Bacon, the provocative case lies not in the germs.

Typhus is the one exanthem, the only one, about which it seems safe to argue: we know probably as much about it as can be known till some perfectly new method of research is opened to us for all the others, or at least until one of our present clumsy methods is very much altered for the better.

The Baconian method of research involves a consideration of *all* the leading features of the subject matter. The ordinary method of what we now call scientific research is to lay hold of one fact, ride it to death, then to ignore all others.

I think it will be admitted that medical students, like students of all other human arts, are apt to swear upon the words of their masters and very faithfully reproduce their views.

During the term of my curriculum one of our stock subjects of debate was the question as to the nosological separation of typhoid (or as it was more usually called "dothen enterite") and typhus fevers. The most enlightened of our teachers put the typhoid cases in his general wards with the result that they nearly all recovered. Another of the old school put them in the fever wards with the result that they often died of the second disease when convalescent, or almost so, from the first. He overlooked the one great fact, which was patent even to my juvenile mind, that a patient with typhus was either dead or practically well on the fifteenth day of his attack, whereas the fatal symptoms of the typhoid might not supervene till the third or even the fourth week. Since then typhus has been studied with more satisfactory results, and a few of these show that though it was of old and is equally now certain that it is conveyed by the impress of a germ, or poison having the life history of a germ-producing organism, very analogous in its proceedings to the germ of yeast, yet that this organism is perfectly inert when distributed on a soil not responsive to it. The cause of the disease is not the germ or organism arising from it. The cause of the disease is simply over-crowding of population beyond a point ascertained with an accuracy like that of the density bulb or the thermometer in the brewer's vat.

As clinical clerks and resident medical officers in the old infirmary of Edinburgh we know this quite well. Given a case of hemorrhagic typhus, or one in which subsultus tendinum occurred at once, we knew within a narrow limit of error from what part of the city it came. We knew that so many hours' work in the fever wards would give us a headache, and that unless we had our evening's spin round Arthur's Seat our six months' appointment would not pass without our falling permanently victims to the disease. In one of the sessions there were fifteen out of twenty residents in the three great centers of typhus in Scotland, Greenock, Dundee, and Edinburgh, fell victims to the disease, and eight of them died. So awful was this that appointments were made afterwards only of men who had already gone through the ordeal; yet the disease did not spread out of its nests.

The eminent medical officer of the City, Dr. Littlejohn, has solved its riddle, and has practically demolished the disease. He began its study about 1845, and he tells us that in the great outbreak of 1847-8 there occurred an outbreak of which over 6,000 cases were treated in the infirmary alone, and how many outside can never be known. The mortality was awful, it can only be guessed at, probably being not less than 20 per cent.

The outbreak, to use Dr. Littlejohn's own words, "so frightened fathers of intending students and general visitors, that Edinburgh as a seat of a medical school, and as a residential city, was nearly destroyed." It acquired the name of the "fever city," a cognomen amply justified, seeing that at least 15 per cent. of its population were attacked within two years.

Active and energetic steps were taken, huge blocks of tenements were destroyed, dense populations were scattered and wide boulevards cut through the worst parts of the old town, and no such outbreak has since occurred. But the history of the extinction of the disease has been gradual, for such work as had to be done was hardly the work of one generation only. In 1857-8 the number of cases known fell to 139. In 1863 there were fifty-seven deaths representing a number of cases to be given by calculation as about 350.

In 1879 the notification Act came into force, and the exact number of cases as well as the deaths can be given with great accuracy. The number fell steadily. In 1889 there were forty-three cases with nine deaths; in 1892, eighteen cases with three deaths; in 1893, six cases with one death, and this year, so far, there has not been a single case reported. (Two cases reported since this was written).

But this is not all, by any means. The fever districts were in the old town, the regions of Cannongate, Trongate, St. Giles and Cornmarket, having in 1861 an average population of 220 to the acre. By 1881 this was reduced to 166 persons to the acre, the interval representing the time of the chief clearness of the infected area.

The worst cases we had when I was a student were from the high lands of the Tron, where the population then was over 300 to the acre, and some closes in the Grassmarket where it probably was higher. Now the average population has been reduced to less than half, and the very worst places have been completely emptied and typhus has disappeared. Not a case of typhus has ever been known in Birmingham where no part has ever been populated to such an extent, and practically typhus is unknown wherever the population is below 150 to the acre, and it occurs with certainty when it is over 200. Its occasional manufacture by temporary over-crowding in army barracks, in ships, prisons, etc., has been illustrated over and over again till we are forced to the conclusion that the poison, whatever it is, is the result of the same cause as that which gives it its powers.

It is probably the germ of some very ordinary fungus, sporting with deadly growth from the pabulum afforded by the crowd, a suggestion made to me by the late Charles Darwin. At any rate, in this case the fulfilment of Bacon's canon is complete. The phenomenon, a population above a certain density, is always present when the effect, typhus fever, is present. It is universally absent when typhus fever is absent; and the effect, typhus, varies in degree with the degree of over-crowding. The phenomenon, over-crowding, is, therefore, the form in question, "the cause of the given fact or attribute." Littlejohn's facts and figures prove the law of the appearance and existence of typhus, the law of its variation, and they have established the fact of its extinction. You may as well establish a germ theory for this awful disease as for a leg of cold mutton.

Not only is the argument from etiology one of the most complete and perfect kind, but it is supported to a most remarkable degree by many clinical facts. Thus Russell, Murchin, Christison and Wilson all agree upon certain facts (and no one has even disputed them) that typhus arises *de novo* upon appropriate provocation. Its germ is, therefore, certain to be a sport, forced by the conditions so well described by Howard, and we should expect that the conditions of sport being removed it would speedily lose its temporary malignity and return to its ordinary and probably quite harmless form. This is entirely supported by the fact insisted upon by all authors, that the contagion of typhus does not carry far, and is completely and speedily killed by cleanliness and an abundant air supply. Murchison lays special stress on the small risk to attendants on the fever-stricken if these conditions are fulfilled.

All these facts are wholly contradictory of a "germ theory;" for even if a "germ theory" were accepted in this case we should immediately point out that the germ was only a temporary existence, and that we have an already well ascertained cause of its existence. It, therefore, must disappear from the argument, save as a mere stage of a process.

The facts of no other disease of the zymotic class are sufficiently well known yet to form the basis of complete argument, and we shall not arrive at these necessary facts by mere germ hunting, however interesting and important an avocation it may be.

I now come to the crux of my discussion, the application of the so-called germ theory to the practice of surgery, and to an explanation by it of the want of success in surgical practice, which ought to be and has been often and most successfully laid out on other grounds altogether; and to an explanation of modern success and advances in surgery alike erroneously attributed to the "germ theory" and the so-called antiseptic surgery.

I must again remind you of a fundamental rule of logic, that "it is absurd to assert that to be a cause which can be at the most only one of several factors, especially when it can be shown that it is by no means the most important in the extent of its application." Indeed it must be self-evident that any factor which would be important enough to constitute of itself a "theory of disease" must be not only of universal application, but when it is one of a series of factors it must be overwhelmingly the most important.

The great fact of typhus, for instance, as Littlejohn shows overcrowding, is universal and necessary, and ever present. It is as the apodyctic logicians would say, "το καθὸλον" the universalizing element, bringing all others under it, and the "το ϛυλλὸγον" impossible without it. All outside it is mere dialeticism and may be disregarded.

But we cannot treat the surgical division of my subject in this way, for we do not know a universalizing element, though there is one, the same as in typhus, which comes very near indeed.

But the logician has many weapons, and Euclid is full of logical devices. He asks you to believe that if two circles have the same radius they must have the same diameter, circumference, and included area. Provided again, there is not a fourth dimension in space, he proceeds to prove this proposition to you by asking you to assume that it is not so. You super-impose your two circles, and by the construction of a pair of triangles Euclid shows you the true state of affairs by a *reductio ad absurdum.* The true bearing and marvelous applicability of this method of reasoning generally departs with our school days, and its application is entirely absent in the literature of medicine and surgery—else half our text-books were never written.

In byegone times there used to be a very prevalent, and even now there is a far too prevalent disease of protean form which infested, and does still infest, the surgeon's work. We used to call it surgical fever, pyemia, sapremia, and now it is called septicemia—all of which names were coupled with certain doctrines, about which I shall not trouble you. It always was more or less closely associated with views about inflammation. A doctrine of the past generation was that inflammation was *sthenic* and *asthenic*, and that our present malady was associated with the latter form. Then came the discovery of the germ factor in the process of putrefaction, and the explanation of the methods of sterilization, and a false conclusion was built up concerning surgical practice, that the ill results were due to the entrance into wounds of the germs of putrefaction. Then a few special, or at any rate special-looking germs, were identified as the particular germs to be dealt with, but unfortunately for them Bacon's inevitable law throws them out of court. The far more logical position was the assumption that the ever and everywhere present common or garden germs were the cause of the trouble, and by far the most logical method of dealing with them was the spray. It looked so logical, and it was imposing that there is no wonder it rushed headstrong into popularity.

I christened it "The Royal Road to Surgical Success" for two reasons. First it promised so much, being in itself so easy, and it got its inventor a title by favor of royal admiration.

I laughed at and ridiculed it, and now it is pretty well done for, and to the detriment of nobody but the surgical instrument makers.

But that very spray was the cause and means of a discovery by Lister so great that he deserved everything he got in the way of honors, though he did not understand it at the time. I don't think he yet understands the greatness of the discovery he made. I have never tired of pointing it out, but I have never been fully listened to; and when I hailed with triumph the complementary discovery of Hamilton and sponge grafting, I shouted to deaf ears.

Lister's discovery was like all great things, very simple! It was only this, that a blood-clot may and under certain circumstances does retain real life; or at least it is so far removed from complete death that if aided by preservatives it will increase in vitality and be even finally, as we say, organized.

He filled a big hole in a man's heel with protected blood-clot: he kept out the common or garden germs and that blood-clot got organized and filled up the hole. Of course it must not be forgotten that all these phenomena were known in domestic science for centuries before, but their surgical importance was not recognized: and certainly Lister's discovery was new in so far that it referred to blood-clot not covered by skin or other living tissue, but directly exposed to air—air altered and deprived of its dangerous elements. Then comes Hamilton who showed that material actually dead—dead beyond all question—could be so prepared as to adapt itself to tissue of the feeblest kind, granulation cells, could be used by them as a temporary endoskeletal arrangement and removed by them by the common process of absorption when they were no longer in need of it.

This led to many practical results which do not concern me now. The two facts however are so valuable in countless ways that though they are all that is left of the original germ theory as applied to surgery, it was worth all the worry and trouble to get them. Immense efforts have been made to explain them away—

because they have become real stumbling blocks to the real earnest germ-hunter and he would gladly have seen them upset. The last and perhaps most skilful is the phagocyte hypothesis. But as phagocytes cannot move through a blood-clot they do not meet the one great fact of Lister which has stood and still stands against all attacks.

The misfortune is in that he did not read his discovery aright. He concluded that what could make the difference between a blood-clot organizing and one breaking down in decomposition, the ordinary germs of decomposition, were enough to break down and destroy the union of a stump, and finally life itself, after surgical operations; and hence he devised his innumerable and most ingenious contrivances.

He knew from his laboratory experiments that probably a single spore was quite enough, if admitted to a sterilized flask to set all its appropriate contents into a condition of putrefaction; and permeated by a false analogy he concluded that a single germ was equally potent in a surgical wound. He believed that the two circles had equal radii, and that therefore the diameters and other attributes would be the same, but it was not so. At any rate the resulting triangles constructed on the radii did not superimpose. For example, the wounds of shaving were open to germs equally with those of the surgical knife. I have no doubt being a very clumsy barber I have implanted myriads of these germs with fresh wounds in my skin, over and over again, yet no septic results have followed in me nor in the myriads of my fellow-sufferers. But Lister's more logical followers did not see things in his light, and they pushed their arguments everywhere and followed it by their devices.

Thus in a recent number of the *American Journal of Obstetrics* a certain Dr. Allen tells us that he divides the umbilical cord about two and one-half inches from the abdomen, wipes it and the cord with a bichloride solution, firmly secures it with a sterilized ligature, and touches the cut end of the cord with a bichloride tabloid.

Sterilized gauze, sterilized bandages, and a properly sterilized cradle and nurse secure the recovery of the germ endangered baby. Dr. Allen winds up with this final precaution, "Of course the surgeon should prepare his hands as for a formal operation," and no doubt a formal operation fee is charged after the danger is all over.

Here is a case of *reductio ad absurdum*, both in a popular and logical sense. He omits the major premises of his arguments, which would be of course that by his ridiculous proceedings he has saved the lives of countless babies. But his position is the inevitable and wholly reasonable conclusion of an adaptation of this so-called germ theories to the practice of surgery. But I super-impose my circle on his and find the radii and all features are the same in that his factors are out of court, and the verdict is *reductio ad absurdum.*

Similarly and more seriously, but with a result quite as absurd, let me take another recent paper by an American surgeon on "Lacerations of the Perineum"—a subject on which I think I am entitled to speak.

I have tried to estimate how many reparative operations I have performed on the perineum, and I find that it cannot be much short of 1,500, as in one year I have records of 226, whilst for many years I kept no record of them at all, and do not now. If we take them at a thousand it will be enough for my purpose. I have all the records of death certificates that ever occurred in my practice, and there are only two deaths associated with that operation in all my work. Both were old women nearly seventy, on whom any operation, however slight, would have been serious, and in one certainly death occurred from a cause, apoplexy, not a result of the operation, not dependent at least on the question now to be submitted. My mortality, in its greatest reckoning, in this operation, is under .02 per cent. My American friend who criticises my proceedings in most unintelligent and almost unintelligible fashion, gives his own mortality as over 5 per cent. and gives the following rules for operation:

Before and after every operation, wash all instruments in very hot carbolized water, and during every operation keep all instruments immersed in carbolized water. Sutures and ligatures, if of silk and silk-worm gut, are boiled in a dilute solution of corrosive sublimate, and kept immersed in the same until used. Cat-gut is soaked in oil of juniper and preserved in alcohol.

Always bathe denuded surfaces, both before and after approximation, by suture, with a solution of 1 to 10,000 of corrosive sublimate.

Always destroy sponges used in any operation which admits of the possibility of their being contaminated by septic fluid.

Irrigate the vagina daily if there is a discolored or offensive discharge. Prevent the introduction of septic germs from without by covering the vulva with a pad of sublimate gauze, which is to be changed as soiled.

Before every operation let the operator and his assistants cleanse and disinfect, not only the part to be operated upon, but also their own hands and arms, in order that every possible suspicion of septic infection may be removed.

This writer is clearly carrying his convictions to their logical conclusions, but that neither are his convictions based on fact nor his conclusions in the least degree necessary is proved by the fact that my mortality is less than his as one is to 250; yet I never in this operation used a single precaution such as he believes to be essential, nor anything approaching to them.

We have therefore here the so-called "germ theory" and its antiseptic precautions knocked on the head by one of the main features of Bacon's law, that the effect in the most intense degree is constant when the alleged form is absolutely absent, the effect produced without the cause. There may be among you some who would assert that neither division of the umbilical cord nor repair of a damaged perineum are sufficiently serious operations to make any mortality at all. There is then no truth in this alleged germ theory, for the logical deduction of that theory is that the entrance of a germ or germs to any wound whatever is capable of producing systemic septic evils and death. But we must carry the matter further, and into the realms of more general surgery; for has not our President said:

Lister "proved conclusively, and his proofs have been confirmed by many others, that ordinary cleanliness in the dressing of wounds, either made or treated by the surgeon, was not sufficient, but that the use of some kind of antiseptic was absolutely necessary to avoid the risk of septic infection."

Now, sir, you have seen a good deal of my operation work, and I challenge you or anyone to say that they have even seen anything about it save absolute cleanliness. Since the time—nearly twelve years ago—when I ran through a sham Listerism, ending with cold water spray, never has anything in the shape of antiseptic been used in the treatment or dressing of wounds. Yet my published statistics have not yet been either contravened or beaten in their record.

Lister has admitted these two facts, and has pathetically pleaded that there is something in the abdomen which transcends his chemicals. My answer is, it is not so. The abdomen is the only region yet where we have had competing and perfect statistics. I have challenged Sir Joseph over and over again to produce his operation statistics, but with a lordly indifference, or something else, he pays no attention to the challenge. We are constantly hearing from his followers as to the marvelous improvements in results obtained under each and every new phase of the details as they come out; but before they are six months old they are pronounced failures and something new has its turn. The last of all of these numerous phases is the disuse of all chemical destroyers of germs and the abandonment of "antiseptic surgery," and the adoption of "aseptic surgery," the perfect cleanliness which I have been preaching for years; and, forsooth, this is then the "newest Listerism."

But in order to bring logic to bear in the discussion again, let us go back a generation—I have seen it out all but two years, for I am now in my twenty-eighth year of practice—and see what major factors have contributed to surgical success, if success there has been.

First of all, has there been success in the sense that what we may call *coarse* surgery is more successful than it used to be. I do not use this word at all with any disdainful intent; I only mean the class of surgery anybody may do, such as amputations. The finer surgery, such as ophthalmic work, operations for hernia, ovariotomy, tying of arteries, etc., depend so much on the personal skill of the operator that they cannot with success be used in such an enquiry. Besides, in general hands they are not numerous enough. Amputations, especially those for disease, form by far the best test. But can we find them so as to put them up as a test? I think not; certainly I have failed to find them, and the writer who puts them forward, would do infinite service to surgery.

Let me see a list of 500 amputations below the knee for disease, done under fairly good circumstances, and without any of Lister's dodges; and another list of 500 under equally good circumstances, but under the completed system of germicides last announced, and most believed in, and we might get some notion of the value of the latter. The mortality of the second list would have to be less than 5 per cent. before we accept it as worthy of what it pretends to be, for that mortality is proved to be nearly the normal mortality of the operation in question and all mortality above it is an illegitimate excess.

As I look back at my early experience of surgery my wonder is that I ever stuck to it. In Edinburgh, during my pupilage from 1861 till 1866, I saw some thirty ovarian and other abdominal tumors removed without one single recovery, and I left the land of my birth with one fully made resolution, that I would never open an abdomen. In Edinburgh, if I saw an amputation of the thigh in the Old Infirmary on a Wednesday, there was a strong probability that in the following week I would see the bared bone sticking up through the anterior flap.

If a breast was removed, an erysipelatory reddening of the flaps would very probably occur on the third day and be half way round the chest before the week was out, the wound gaping, and everything going to the bad. Erysipelas indeed was rampant.

I left Edinburgh and have been engaged continuously ever since, with very slight interludes indeed, in making wounds, till they have mounted up to many thousands, and I have never seen a case of erysipelas in my own practice, not one. This, of course, gives exactly the other side of the picture, and an enormous improvement. It does not in any way belong as a result to chemical germicides, because I don't use them, and never did, save in the few months of the experiment above alluded to.

In what lies the improvement? The answer is simple; it lies in separation of patients; plenty of cubic space and fresh air. The road to this conclusion was opened by Simpson, in his book on "Hospitalism;" he shows that the mortality of amputation below the knee for diseases diminishes, as did that of all amputations, just in proportion to the size of the hospital, and the crowding of the patients which naturally resulted.

This was confirmed by my own research and independently by others. It would then clearly need, according to Bacon's canon, to be shown that by chemical antiseptics the mortality of this operation is brought down in the largest class of hospitals lower than it is in the smallest before the slightest credit could be given to it. Since then most of our large hospitals have been rebuilt with the most extensive improvements, and it is truly pitiable to look at the records of mortality of some that have not. Amongst the latter is King's College Hospital, and the records there (I have had a private glance at some of them) are so bad that they dare not publish them; at least, I have challenged them to do so for the last sixteen years and they have not done so. Even in cases where these large hospitals have not been rebuilt, their surgical overcrowding has been remedied by the building all round them of small hospitals, no better example of this being possible than the change effected in our own city within these last twenty-five years.

The conclusion of the argument is that this surgical scourge is but another form of typhus—a view which is by no means a novelty—the result of over-crowding wounded and discharging surfaces. The results of experiments—generally on a scale of ghastly magnitude and with dreadful results—have shown that without over-crowding the effect is rarely present; with overcrowding it always is: and above all things the experiments of irritating surgery have shown that just in proportion as the intensity of the cause, so is the effect varied. All the clinical facts spoken of, concerning typhus, bring out the analogy with increasing strength.

It was Simpson who cried out after Howard against over-crowding in hospitals. It was Simpson who cried out most loudly for better ventilation and cleanliness, and against the use of dirty hand and sponges.

It was he who taught after Semelweiss the doctrine of contagium vivum in midwifery, and appealed for the aseptic treatment of lying-in women. By anesthetics it was Simpsou who made all our modern surgery a possibility.

He has been dead hardly these five and twenty years, and all this splendid work is as much forgotten as if it had never been done, and the glorious progress which has come out of it is given to a theory which is no theory at all but a phantasm, to a system which has been proved an inconstancy and a broken reed—a thing which yields at every blast either to scholastic logic or eclectic experiment.

---

Grassow (*Centralbl. f: Gynäk.*, No. 43, 1894,) recently exhibited at a German medical society a parovarian cyst which had contained twenty-five pints of fluid. The patient had gone to the end of her eleventh pregnancy. The tumor caused a marked distortion of the child's skull. Fourteen days after delivery, ovariotomy was performed. The bulky uterus, in imperfect involution, gave great trouble to the operator. The base of the cyst, being sessile, was with difficulty detached; the pedicle required very careful ligature, or rather suture. Grassow concludes that, whatever may be said for ovariotomy during pregnancy, it is not right, save when urgent symptoms appear, to perform that operation after delivery till involution is complete.—*British Medical Journal*, November 10, 1894.

